# M2 Tumor-Associated Macrophages and Microvessel Density at the Invasive Front of Resected Gastric Adenocarcinoma: A Clinicopathological Study

**DOI:** 10.3390/cancers18060904

**Published:** 2026-03-11

**Authors:** Mariusz Szajewski, Maciej Ciesielski, Aleksandra Ciarka, Wiesław Janusz Kruszewski, Łukasz Fręchowicz, Dariusz Nałęcz, Piotr Kurek, Przemysław Miłosz, Daria Jarzębowska, Jacek Zieliński

**Affiliations:** 1 Department of Oncological Surgery, Gdynia Oncology Centre, 81-519 Gdynia, Poland; maciej.ciesielski@gumed.edu.pl (M.C.); wieslaw.kruszewski@gumed.edu.pl (W.J.K.); lfrechowicz@szpitalepomorskie.eu (Ł.F.); piotr_kurek@gumed.edu.pl (P.K.); 2 Department of Oncological Surgery, Faculty of Health Sciences with the Institute of Maritime and Tropical Medicine, Medical University of Gdansk, 80-210 Gdansk, Poland; 3 Department of Pathomorphology, Faculty of Medicine, Medical University of Gdansk, 80-210 Gdansk, Poland; aleksandra.ciarka@gumed.edu.pl; 4 Department of Otolaryngology and Maxillofacial Surgery, St. Vincent de Paul Hospital, Pomeranian Hospitals, 81-348 Gdynia, Poland; dnalecz@szpitalepomorskie.eu; 5 Student Scientific Circle, Department of Pathomorphology, Medical University of Gdansk, 80-210 Gdansk, Poland; przemyslaw.milosz@gumed.edu.pl; 6 Department of Clinical Pathomorphology, University Clinical Center in Gdansk, 80-214 Gdansk, Poland; d.jarzebowska.340@studms.ug.edu.pl; 7 2nd Division of Radiology, Faculty of Health Sciences with the Institute of Maritime and Tropical Medicine, Medical University of Gdansk, 80-210 Gdansk, Poland; jaziel@gumed.edu.pl

**Keywords:** gastric cancer, tumor microenvironment, M2 tumor-associated macrophages, CD163, microvessel density (MVD), angiogenesis

## Abstract

**Background/Objectives**: Gastric cancer (GC) remains a major cause of cancer-related deaths worldwide. The tumor microenvironment, particularly tumor-associated macrophages (TAMs), plays a key role in tumor progression and angiogenesis. M2-polarized TAMs are considered pro-tumorigenic; however, their prognostic significance and relationship with microvessel density (MVD) at the invasive front of GC remain unclear. This study evaluated the prognostic significance of CD163^+^ M2 TAMs and MVD at the invasive front of gastric adenocarcinoma. **Methods**: A retrospective analysis was performed on 106 patients who underwent radical R0 surgery for GC without neoadjuvant therapy. Immunohistochemistry using CD163 and CD34 antibodies was applied to quantify M2 TAMs and MVD at the invasive front. Median values were used as cut-offs. Associations with clinicopathological features were analyzed, and survival was assessed using Kaplan–Meier and Cox regression models. **Results**: The mean number of CD163^+^ M2 TAMs was 70, and the mean MVD was 33. A significant positive correlation between M2 TAMs and MVD was observed (*p* = 0.001). Higher M2 TAMs infiltration was associated with deeper tumor invasion (pT) and showed a trend toward advanced pTNM stage. In univariate analysis, neither M2 TAMs nor MVD was associated with survival. Multivariate analysis identified pT (*p* = 0.02) and MVD (*p* = 0.03) as independent prognostic factors, with increased MVD associated with improved overall survival. **Conclusions**: CD163^+^ M2 TAMs are linked to angiogenesis at the invasive front of gastric cancer and correlate with tumor invasiveness. Increased MVD may represent an independent favorable prognostic marker, highlighting the biological importance of the invasive tumor front.

## Simple Summary

Gastric cancer is associated with poor survival, and improved understanding of the tumor microenvironment may help refine prognosis and guide future therapies. Among immune cells, tumor-associated macrophages (TAMs) play an important role, particularly M2-polarized macrophages, which promote tumor growth, invasion, and angiogenesis. In this study, we analyzed tissue samples from 106 patients who underwent radical R0 surgery for gastric cancer. We focused on the tumor invasive front and assessed the number of M2 macrophages (CD163^+^) and microvessel density (MVD). Higher numbers of M2 macrophages were associated with increased angiogenesis and deeper tumor invasion. Although M2 macrophages were not independent predictors of survival, higher microvessel density was independently associated with better overall survival. These findings suggest that the tumor invasive front is a biologically distinct region where interactions between macrophages and blood vessels influence tumor behavior and may have prognostic relevance in gastric cancer.

## 1. Introduction

Gastric cancer (GC) is the fifth most common malignancy and the fifth leading cause of cancer-related death worldwide [1]. Chemotherapy used as an adjunct to surgical treatment is considered to be of limited effectiveness in this disease [2]. Gastric cancer arises as a consequence of accumulated genetic alterations; however, its development and progression depend on interactions with factors present in the surrounding tumor microenvironment (TME). Consequently, the TME has become the focus of intensive research as a potential target for anticancer therapies [2,3,4,5,6,7,8,9,10,11].

The tumor microenvironment is enriched with immune system components, among which tumor-associated macrophages (TAMs) represent a dominant population, accounting for up to 50% of immune cells within the TME. TAMs originate from circulating blood monocytes as well as from tissue-resident macrophages of embryonic origin, with a small proportion derived from the yolk sac, liver, and spleen [6,12,13,14]. These relatively long-lived cells are characterized by remarkable functional diversity and plasticity [4,6,8,10,15].

TAMs are generally classified into M1 and M2 phenotypes. M1 macrophages exhibit pro-inflammatory and antitumor activity, whereas alternatively activated M2 macrophages promote tumor invasion and metastasis through the secretion of anti-inflammatory cytokines, matrix metalloproteinases, and pro-angiogenic factors. Outside the TME, M2 macrophages reduce inflammatory responses, promote angiogenesis, and participate in connective tissue remodeling during wound healing. Within the TME, however, the same functions may facilitate tumor progression [4,6,10,12,13,16]. M2 macrophages support cancer development and progression through the activity of cytokines such as interleukin-10 (IL-10) and transforming growth factor-β (TGF-β) [5,6,8,9,12,14,15,16,17]. The exceptional plasticity of TAMs allows them to adopt either protumorigenic M2 or antitumorigenic M1 phenotypes in response to environmental cues [6,10,11,13,15].

Numerous studies have demonstrated a positive association between increased M2 macrophage infiltration in primary gastric tumors and poor prognosis, as well as adverse clinicopathological features such as tumor size, depth of invasion, and the presence of metastases [5,6,14,17,18,19,20,21,22], although conflicting results have also been reported [12,23]. Heterogeneous distribution of M2 macrophages within the tumor mass has been described, with consistently higher infiltration observed at the tumor invasive front, not only in gastric cancer [12,21,24,25,26]. Increased M2 TAM density at the invasive front is commonly associated with unfavorable prognostic features in gastric cancer [12,20,21]. Enhanced M2 macrophage infiltration is frequently accompanied by increased microvessel density (MVD) within the tumor [6,11,20,24,26]. In a meta-analysis based on a cohort of 4094 gastric cancer cases, increased microvessel density (MVD) was shown to be associated with poorer prognosis in terms of both time to recurrence and overall survival. MVD measurements were not stratified according to specific regions within the primary tumor [27]. Similarly to M2 tumor-associated macrophages (M2 TAMs), MVD has been demonstrated to reach its highest values at the invasive front of the tumor; however, the prognostic value of MVD specifically in this region has not yet been clearly established [20,24]. The prognostic significance of MVD at the invasive front of primary gastric cancer, as well as its relationship with established clinicopathological prognostic factors, therefore, remains to be elucidated.

## 2. Materials and Methods

### 2.1. Patients and Study Design

This retrospective study included 106 patients who underwent radical R0 surgery for gastric adenocarcinoma at the Department of Oncologic Surgery, Medical University of Gdańsk, between 2006 and 2013. None of the patients received neoadjuvant therapy prior to surgery. The study was approved by the local Bioethics Committee (NKBBN/427/2014). Due to the retrospective nature of the study, the requirement for written informed consent was waived.

The minimum follow-up period for surviving patients was 61 months. During follow-up, 44 patients died, and all deaths were cancer-related. The mean overall survival for the entire cohort was 64 months, with a median survival of 69 months. Clinicopathological characteristics of the study group are summarized in Table 1. In four patients, single liver metastases undetected during preoperative diagnostics were identified intraoperatively and resected synchronously with gastrectomy. Patient age was calculated at the time of surgery. Tumor stage was determined according to the AJCC pTNM classification, 8th edition [28]. In the tumor location category, cardia refers to Siewert type III tumors [29], whereas the category “other location” includes tumors located in the gastric body, along both curvatures, and in the pyloric region.

### 2.2. Immunohistochemistry

Immunohistochemical staining and microscopic evaluation were performed at the Department of Pathomorphology, Medical University of Gdańsk. Gastric cancer specimens were processed according to standard histopathological protocols. Immediately after surgical resection, tissue samples from the tumor invasive front were fixed in 10% neutral buffered formalin for 48 h at room temperature. Following fixation, samples were dehydrated through a graded series of ethanol solutions (70%, 80%, 96%, and absolute ethanol), cleared in xylene, and embedded in low-melting-point paraffin.

Paraffin blocks were sectioned into 4 μm-thick slices using a sliding microtome (LEICA Leica SM2010 R; Leica Biosystems; Heidelberger Straße 17-19, 69226 Nussloch, Germany). Sections were mounted on glass slides for immunohistochemical detection of CD34 and CD163.

The following primary antibodies were used:

- CD34: DAKO FLEX, Monoclonal Mouse Anti-Human CD34 Class II, clone QBEND10, ready-to-use (Dako Denmark A/SProduktionsvej, 422600 Glostrup, Denmark). Staining was performed using a DAKO Autostainer Link 48 according to the manufacturer’s standardized protocol.
- CD163: Mouse monoclonal antibody (clone MRQ-26; Roche Diagnostics, 9115 Hague Rd, Indianapolis, IN, USA). Staining was performed using a BenchMark ULTRA system (Roche) according to the manufacturer’s standardized protocol.

Microscopic evaluation was performed independently by two experienced pathologists (R.P. and A.C.) using an Olympus BX43 light microscope (Ishikawa-machi, Hachioji-shi, Tokyo, Japan).

**Figure 1:**
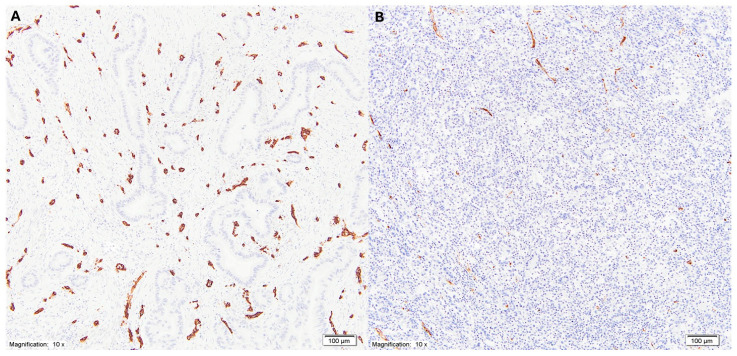
(**A**) Histological image showing invasive tubular gastric adenocarcinoma characterized by an abundant stromal vascular component. Numerous blood vessels are present within the tumor stroma, with endothelial cell lining highlighted by CD34 immunohistochemical staining (magnification ×10). (**B**) Histological image showing invasive tubular gastric adenocarcinoma with sparse stromal vascular component. Only a limited number of blood vessels are present within the tumor stroma, with endothelial cell lining highlighted by CD34 immunohistochemical staining (magnification ×10).

Microvessel density (MVD) was assessed in the tumor invasive front using anti-CD34 monoclonal antibodies according to the method described by Weidner et al. [30]. Briefly, areas with the highest density of CD34-positive vessels (hot spots) were identified at low magnification (×100). Microvessels were then counted at high magnification (×200) within three selected hot spots, and the mean value was calculated as the MVD (Figure 1).

Similarly, CD163-positive macrophages were evaluated in the tumor invasive front according to the recommendations of the International Immuno-Oncology Biomarker Working Group for the assessment of tumor-infiltrating immune cells in solid tumors [31]. The invasive front was defined as the deepest area of tumor infiltration into adjacent non-neoplastic tissue, identified on routine hematoxylin and eosin sections as the tumor–host interface (a 1 mm-wide zone centered on the border separating malignant epithelial nests from adjacent host tissue). The same definition was applied uniformly across all pT categories, and no Lauren subtype-specific modification of the invasive margin definition was introduced. The three high-power fields were systematically selected along the 1 mm invasive margin, following identification of this standardized zone at low magnification. No random selection and no hotspot-driven selection were performed, in accordance with the Working Group recommendations [31].

**Figure 2:**
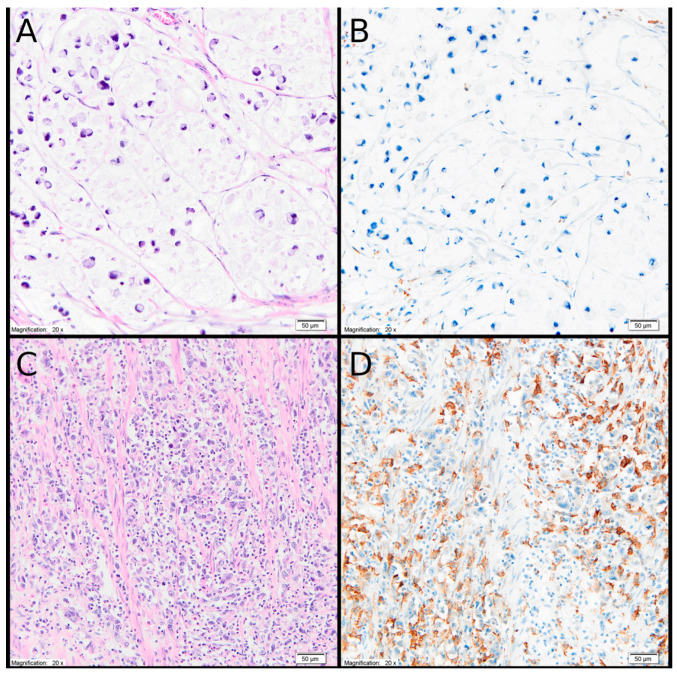
(**A**) Mucinous gastric carcinoma composed of signet ring cells, demonstrated on hematoxylin and eosin (H&E) staining (original magnification ×20). (**B**) The same case as shown in panel (**A**), stained with anti-CD163 antibody, showing no detectable M2 macrophages (original magnification ×20). (**C**) Invasive high-grade gastric adenocarcinoma with an accompanying dense inflammatory infiltrate within the tumor stroma, visualized on H&E staining (original magnification ×20). (**D**) The same case as shown in panel (**C**), immunostained with anti-CD163 antibody, revealing a marked abundance of M2 macrophages (original magnification ×20).

Microscopic evaluation was performed independently by two experienced pathologists blinded to clinical outcomes. In cases of discrepancy, slides were reviewed jointly to reach consensus. Quantitative assessment was performed manually by counting CD163-positive cells at ×400 magnification in three high-power fields (HPFs). The final result was expressed as the mean number of positive cells per HPF. Cell counting was performed without preferential selection of areas with increased cell density (hot spots). Areas of necrosis and regions affected by crush artifacts were excluded from the analysis (Figure 2).

For statistical analysis, tumors were categorized into groups with low (L) or high (H) MVD and low (L) or high (H) numbers of M2 TAMs. Due to the lack of established, biologically and clinically validated cut-off points for M2 TAM and MVD in the analyzed population, as well as the exploratory nature of the study, the median was used as an objective and reproducible method of dichotomization. This approach minimizes the risk of model overfitting that may occur when thresholds are determined based on ROC analysis in cohorts with limited sample sizes.

### 2.3. Statistical Analysis

Statistical analyses were performed using Statistica software (version 13; TIBCO Software Inc., Palo Alto, CA, USA). Associations between M2 TAMs counts and MVD were assessed using the Mann–Whitney U test and Pearson’s correlation coefficient (r). Relationships between clinicopathological parameters of gastric adenocarcinoma and MVD or M2 TAMs were evaluated using the Mann–Whitney U test or Kruskal–Wallis analysis of variance, depending on the number of categories in the grouping variable.

Overall survival was analyzed using the Kaplan–Meier method, and differences between groups were compared using the log-rank test. Multivariate survival analysis, including MVD and M2 TAMs as continuous variables, was performed using the Cox proportional hazards regression model. The proportional hazards assumption was verified using Schoenfeld residual plots. Covariates were selected a priori based on their documented clinical and biological significance, rather than using stepwise statistical selection. The model included: the T feature (2 categories: T1 + T2 vs. T3 + T4), the N feature (2 categories: N− vs. N+), vessel density (continuous variable), and the number of macrophages (continuous variable). T and N features were included as core, established prognostic factors in gastric cancer, reflecting local tumor advancement and lymph node involvement, respectively. Vessel density and the number of macrophages were included as biologically justified variables, representing the focus of the study and potentially independent components of the tumor microenvironment. Clinical stage was excluded from the multivariable model to avoid collinearity. A *p*-value < 0.05 was considered statistically significant.

## 3. Results

The mean number of M2 TAMs in the analyzed cohort was 70 (median: 58; range: 6–279). The mean microvessel density (MVD) in the analyzed group was 33 (median: 30; range: 7–80). A statistically significant association between the number of M2 TAMs and MVD was demonstrated (*p* = 0.001, Mann–Whitney U test; Table 2). With increasing numbers of M2 TAMs, MVD increased significantly (*p* = 0.03; Table 3).

Table 4 presents the results of the analysis of associations between M2 TAMs and MVD and selected clinicopathological parameters of gastric cancer in the analyzed cohort. A significant association was observed between the number of M2 TAMs and the depth of tumor invasion (pT), as well as a borderline association between the number of M2 TAMs and the pathological stage of disease according to the pTNM classification. The highest numbers of M2 TAMs and MVD were observed in tumors with pT3 invasion depth and in patients with pTNM stage III disease. No significant associations were found between MVD and clinicopathological parameters of gastric cancer in the analyzed group.

In univariate analysis, no significant associations were observed between either the number of M2 TAMs or MVD and patient prognosis (Table 5). Multivariate analysis using the Cox proportional hazards model demonstrated that pT stage and MVD were independent prognostic factors. An increase in pT stage was associated with worse prognosis, whereas higher MVD values were associated with improved prognosis in the analyzed cohort. Specifically, for pT, HR = 3.6; 95% CI [0.19–2.35]; *p* = 0.02, and for MVD, HR = 0.97; 95% CI [−0.05 to −0.002]; *p* = 0.03. For M2 TAMs, the Cox model revealed no significant prognostic impact (HR = 0.99; 95% CI [−0.01 to 0.003]; *p* = 0.2).

## 4. Discussion

Multiple molecular mechanisms underlying the activity of M2-polarized tumor-associated macrophages (M2 TAMs) as promoters of gastric cancer (GC) cell proliferation, growth, invasion, and metastasis have been identified [2,3,5,6,7,8,9,13,15]. Through close interactions with cancer cells, M2 TAMs contribute to the induction of immunosuppression, extracellular matrix remodeling, angiogenesis and lymphangiogenesis, and the formation of metastases [5,6,7,9,10,11,15,16,17]. They secrete proangiogenic factors, including vascular endothelial growth factor A (VEGF-A), epidermal growth factor (EGF), tumor necrosis factor alpha (TNF-α), and chemokine (C-X-C motif) ligand 12 (CXCL12) [6,15].

M2 TAMs also induce the production of factors released into the tumor microenvironment (TME) and systemic circulation, which accumulate at distant sites and remodel the local microenvironment into a premetastatic niche that facilitates colonization and progression of circulating tumor cells into metastatic gastric cancer lesions [5,9,14,15]. Furthermore, M2-polarized TAMs promote lymphatic metastasis by stimulating lymphangiogenesis through vascular endothelial growth factor C/vascular endothelial growth factor receptor 3 (VEGF-C/VEGFR-3) signaling, leading to upregulation of VEGF-C production by cancer cells, immune cells, and other TME components [3,6,16,17].

Beyond promoting tumor cell proliferation, M2 TAMs facilitate local invasion, suppress antitumor immune responses, induce chemoresistance, modulate metabolic pathways, and interfere with the local gastric microbiota. Consequently, they are recognized as markers of poor prognosis in many malignancies, including gastric cancer [5,6,9,11,12,15,17]. It is well established that gastric tumors exhibit marked intratumoral heterogeneity with respect to macrophage density and the relative proportions of M1 and M2 phenotypes; however, M2 macrophages tend to predominate in more advanced tumors [6,12,18].

More than a decade ago, Pantano et al. [23] retrospectively evaluated the relationship between M1 and M2 TAMs and survival outcomes in 52 patients undergoing radical surgery for gastric cancer. Similarly to our findings, they did not observe an association between M2 expression and long-term survival. However, multivariate analysis revealed that the M1/M2 macrophage ratio was an independent prognostic factor, with higher ratios indicating better outcomes. Immunofluorescent staining using CD163 antibodies was employed to identify M2 macrophages, as in our study. The authors did not specify the tumor regions from which samples were obtained for macrophage density assessment. Notably, the CD163 antibody clone MRQ-26 used in our study is among the most widely applied immunohistochemical markers for M2 macrophage identification [12].

In a 2016 study, Park et al. [20] analyzed 113 gastric cancers and demonstrated that higher numbers of CD163+ TAMs (i.e., M2) in both tumor stroma and invasive margins were positively correlated with increasing tumor size, depth of invasion, pTNM stage, lymph node metastasis, and lymphatic vessel invasion. High M2 TAM density in stromal and marginal regions was significantly associated with worse prognosis. Cut-off values for immunostaining were determined using receiver operating characteristic (ROC) curves, accounting for sensitivity and specificity in tumor progression. High M2 expression thresholds were set at 102 in the invasive front, 90 in the stroma, and 77 within the tumor core, while the MVD cut-off was set at 15 using CD105 as a vascular marker. The authors also demonstrated a significant association between increased vascular density and higher M2 TAM counts regardless of tumor location, suggesting a direct role of M2 macrophages in angiogenesis. These findings are consistent with our results regarding the invasive front of gastric cancer.

In contrast to Park et al., we did not observe a direct impact of M2 TAM density at the invasive front on patient prognosis. Nevertheless, the association between higher M2 counts and deeper tumor invasion (pT according to AJCC TNM classification), as well as the trend toward correlation with advancing disease stage, indirectly supports a relationship between M2 TAM density at the invasive front and gastric cancer prognosis.

A meta-analysis published nearly a decade ago by Yin et al. [22], including 19 studies and 2242 patients, showed that total TAM density (combining M1 and M2) in primary gastric tumors lacked prognostic significance. However, high M1 TAM density was associated with improved overall survival (OS) (HR = 0.45, 95% CI: 0.32–0.65), whereas high M2 TAM density correlated with poorer OS (HR = 1.48, 95% CI: 1.25–1.75). Elevated M2 TAM levels were also significantly associated with larger tumor size, diffuse Lauren type, poor histological differentiation, deeper invasion, lymph node metastasis, and higher pTNM stage. The authors emphasized that this was the first meta-analysis to clearly differentiate prognostic outcomes based on M1 versus M2 macrophage expression in gastric cancer. Importantly, M2 expression at the invasive front was not separately analyzed.

In a recent study by Zhang et al. [16] involving 80 gastric cardia carcinomas, immunohistochemical analysis revealed significantly higher M2 TAM expression within tumor tissue compared with regions located at least 5 cm from the tumor margin. Increased M2 expression correlated positively with tumor size greater than 5 cm, invasion depth, and AJCC TNM stage. In our study, limited to assessment of M2 expression at the invasive front, we similarly confirmed associations with tumor invasion depth and observed a trend toward correlation with TNM stage.

Using Stereo-seq technology, the existence of an invasive zone centered on the tumor border, distinct from other tumor regions, has been demonstrated in hepatocellular carcinoma. Within this zone, tumor cells exhibited reprogramming associated with increased aggressiveness, and a marked enrichment of anti-inflammatory M2 macrophages (comprising more than two-thirds of macrophages) was observed. Moving deeper into the tumor, angiogenesis and epithelial–mesenchymal transition (EMT) signatures diminished [24].

The tumor margin differs dynamically from the tumor core, forming an immunological barrier supported by stromal remodeling that facilitates both local invasion and systemic dissemination. Compared with the tumor center, differences involve TME composition, angiogenesis, metabolism, and cellular phenotype, promoting extravasation of circulating tumor cells through newly formed blood and lymphatic vessels at the tumor periphery, with a pivotal role of M2 TAMs. Enhanced angiogenesis at the tumor margin has been documented, characterized by activation of multiple angiogenic pathways exceeding those observed in the tumor core. Moreover, tumor endothelial cells (TECs), unlike normal endothelial cells, actively participate in extracellular matrix remodeling and immune regulation [24,26]. In accordance with international recommendations for immune cell evaluation in solid tumors [31], we performed macrophage quantification as an average assessment along the invasive front and did not focus on vascular hot spots. It is conceivable that restricting macrophage counts to MVD hot spots might have yielded stronger associations with angiogenesis; however, such an approach could bias results toward areas of maximal vascular proliferation rather than reflecting the overall immune microenvironment.

Among the four M2 subtypes (M2a, M2b, M2c, M2d), M2d macrophages—activated by toll-like receptors (TLRs), VEGF, and IL-10—play a key functional role in angiogenesis and cancer progression. Angiogenesis supplies tumors with oxygen and nutrients while facilitating waste removal [6]. Proangiogenic factors secreted by M2 TAMs include VEGF-A and VEGF-C, fibroblast growth factor (FGF), platelet-derived growth factor (PDGF), and angiopoietins 1 and 2 (ANG1 and ANG2). M2 TAMs also secrete proteolytic enzymes that remodel the extracellular matrix, thereby facilitating neovascularization [6,15]. A positive correlation between M2 TAM density, VEGF levels, and MVD has been consistently reported in mammalian tumors [6], which aligns with our findings. Importantly, we additionally identified MVD as an independent prognostic factor associated with improved survival in gastric cancer.

One of the earliest studies demonstrating increased accumulation of M2 TAMs at the invasive front of gastric cancer compared with other tumor regions was conducted by Wu et al. in 2012 [21] on a cohort of 115 gastric cancers. The authors confirmed an association between increased vascular density and elevated M2 expression at the tumor margin, as well as a link between high M2 expression, advanced disease stage, and increased metastatic potential.

A recent comprehensive review analyzing multiple epithelial malignancies, including 5139 gastric cancers, consistently reported higher M2 macrophage density at the invasive front compared with stromal or intratumoral epithelial regions. The authors emphasized that increasing M2 density at the invasive margin was frequently associated with poorer prognosis, including in gastric cancer, although this correlation was often observed only in univariate analyses and not confirmed in multivariate models. Notably, M2 density in tumor regions other than the invasive front was frequently not associated with prognosis. Furthermore, M2 density typically decreased from the invasive front toward the tumor core across various cancer types [12].

In a 2017 meta-analysis by Hong W.G. et al. [27], based on 4094 gastric cancer cases derived from 26 studies (24 from Asia and only 2 from Europe), a significant association was demonstrated between increased microvessel density (MVD) and shorter time to recurrence as well as reduced overall survival. MVD was significantly elevated in tumors of the diffuse type according to Lauren’s classification, in cases with lymphatic invasion, lymph node metastasis, higher pT stage, and more advanced pTNM stage. No association was found between MVD and vascular invasion or tumor grading. CD34 was the most commonly used endothelial marker for microvessel assessment, typically evaluated at ×200 magnification. Importantly, the authors did not stratify the analyzed studies according to the specific tumor region in which MVD was assessed.

In a study published one year later, Shi et al. [32] from China, analyzing a substantial cohort of 170 gastric cancer cases, reported that MVD measured using CD34 at ×200 magnification was not associated with prognosis in either univariate or multivariate analysis. Similar findings were reported by Park et al. [20] from South Korea in 2016, based on 113 gastric cancer cases. Using CD105 as a microvessel marker, they found no significant correlation between MVD and overall survival (OS). Although increased MVD was associated with shorter disease-free survival (DFS) in univariate analysis, this relationship was not confirmed in multivariate analysis. In contrast, Pavlovic et al. [33], analyzing 70 gastric cancer cases and using CD34 as an endothelial marker, demonstrated that increased MVD was significantly more frequently observed in better-prognosis intestinal-type tumors according to Lauren’s classification than in poorer-prognosis diffuse-type tumors.

Further evidence supporting the independent negative prognostic value of increased MVD in primary gastric cancer was provided by the more recent study of Li F. et al. [34] from China, based on 1121 gastric cancer cases. They also demonstrated significantly higher MVD in HER2-positive tumors. Microvessels were identified using CD34 immunostaining. Relying on the prognostic value of MVD assessed by CD34, Liu H. and Zhao K-Y [35], also from China, combined MVD evaluation in endoscopic biopsy specimens from primary gastric tumors with three-phase dynamic contrast-enhanced computed tomography (CT) in the preoperative staging of 106 patients. They showed that CD34 expression together with three-phase dynamic contrast-enhanced CT enabled highly accurate preoperative assessment of tumor invasion depth and lymph node metastasis. In all of the above-cited studies, the specific tumor region in which MVD was evaluated was not distinguished.

The invasive potential of the tumor front—so critical in cancer progression—is shaped by multiple biological processes, including angiogenesis, lipid metabolism, glucose metabolism, and multistep epithelial–mesenchymal transition (EMT). These processes undergo dynamic changes under the active influence of numerous tumor microenvironment (TME) components, including the highly plastic and still incompletely understood polarization of tumor-associated macrophages (TAMs) between M1 and M2 phenotypes, as well as their intermediate forms. TAMs may be readily reprogrammed from an M2-like immunosuppressive state toward an M1-like pro-inflammatory phenotype [6,24,36,37]. Investigators exploring the promising role of metabolic processes in the complex interplay between proliferative activation and immune evasion—hallmarks of malignant adaptation observed in vitro—emphasize the need for confirmation of these findings in animal models or patient-derived xenografts, highlighting the persistent gaps in our understanding [38]. Nevertheless, the review by Chen Y. et al. [37] on metabolic reprogramming in lung cancer clearly demonstrated that enhanced glycolysis, dysregulated lipid synthesis and oxidation, and altered amino acid metabolism collectively contribute to immune resistance.

The biological significance of cholesterol 25-hydroxylase (CH25H) has also been investigated in various malignancies, revealing prognostic differences depending on its expression level. It has been hypothesized that, in gastric cancer, the CH25H–25-HC metabolic pathway may function as a context-dependent nexus between tumor metabolism and immunity, with CH25H expression being associated with poorer prognosis [39]. This complexity makes it difficult to definitively characterize TME dynamics and their consequences based solely on the measurement of selected factors.

It should also be emphasized that commonly used endothelial markers for MVD assessment, including the widely applied CD34, do not allow differentiation between mature and immature vessels, which may be relevant for identifying vessel co-option. Vessel co-option, currently considered a principal mechanism underlying chemoresistance in malignancies, represents a non-angiogenic mode of tumor vascularization in which cancer cells utilize pre-existing blood vessels rather than inducing neovascularization. In addition, vasculogenic mimicry (VM)—a process in which tumor cells form vessel-like, fluid-conducting structures lacking endothelial cells—must also be taken into account [40]. These diverse mechanisms influence tumor perfusion efficiency and effectiveness, thereby contributing to cancer progression and aggressiveness [6,36,40].

In the meta-analysis by Hong W.G. et al. [27], MVD did not significantly correlate with primary tumor size, tumor differentiation, or vascular invasion. Tumor size itself has not consistently emerged as an independent adverse prognostic factor in gastric cancer, as demonstrated in the cohort of 1121 cases analyzed by Li F. et al. [34]. Similarly, Tao X. et al. [41], in a study of 100 gastric cancer cases, found no association between tumor size and prognosis or MVD. It cannot be excluded that increased MVD at the invasive front may provide improved local perfusion, thereby facilitating tumor growth without necessarily reflecting greater biological aggressiveness. This possibility may partly explain the findings of our study, which demonstrated a positive association between increased MVD at the invasive front of primary gastric cancer and improved overall survival.

Despite decades of research on the prognostic value of M2 TAMs and MVD in different regions of gastric adenocarcinoma, their clinical significance remains inconclusive. Uniform cut-off values for M2 TAM and MVD assessment in gastric cancer have not been standardized [5,6,12]. Continued investigation into molecular regulators of M1/M2 polarization and angiogenesis remains essential, rendering this field open to further research [2,3,4,5,8,11].

Our study has several limitations, including its single-center, retrospective design and relatively small cohort size. We evaluated neither the relationship between M2 tumor-associated macrophages (M2 TAMs) nor microvessel density (MVD) at the invasive front and established prognostic features of primary gastric cancer, such as angiolymphatic invasion, perineural invasion, or tumor grading. Additionally, the CD163 marker used to identify M2 macrophages does not fully capture the functional heterogeneity of the TAM population. Nevertheless, our findings demonstrate a novel association between increased MVD and improved prognosis in gastric cancer, warranting further investigation and validation in larger, prospective studies.

## 5. Conclusions

In summary, we confirm a significant association between increased CD163^+^ M2 tumor-associated macrophage (M2 TAM) expression and elevated microvessel density (MVD) at the invasive front of gastric adenocarcinoma. We also demonstrate enhanced CD163^+^ M2 TAM expression in gastric cancers with greater depth of invasion according to the AJCC TNM pT classification, which—together with the observed trend toward higher expression in more advanced disease (*p* = 0.06)—may indirectly indicate an association with poorer prognosis. In contrast, increased MVD at the invasive front of gastric cancer appears to be associated with improved patient outcomes.

## Figures and Tables

**Table 1:** Clinicopathological characteristics of 106 patients with gastric adenocarcinoma.

Parameter		No. of Patients	%
Age (median-62.5 years)	<62.5	53	50
	≥62.5	53	50
Gender	Female	32	30
	Male	74	70
Depth of invasion (pT)	T1 (a + b)	9 (1 + 8)	8
	T2	16	15
	T3	55	52
	T4 (a + b)	26 (26 + 0)	25
Regional lymph nodes (pN)	N0	29	27
	N + (N1 + N2 + N3_a+b_)	77 (23 + 22 + 32_21+11_)	73
Stage (pTNM)	I (A + B)	13 (7 + 6)	12
	II (A + B)	37 (18 + 19)	35
	III (A + B + C)	52 (23 + 19 + 10)	49
	IV	4	4
Histological type (Lauren)	Intestinal	56	53
	Diffuse	22	21
	Mixed	28	26
Location of tumor	Cardia	39	37
	Other location	67	63

**Table 2:** Relationship between MVD (group Low and High) and M2 TAMs (Mann–Whitney U test).

Parameter		M2 TAMs Mean (SD)	*p* Value
MVD (CD34)	L group < 30	55 (32)	0.001
	H group ≥ 30	83 (50)	

**Table 3:** Correlation between M2 TAMs and MVD (Pearson’s r coefficient).

Parameter	MVD (CD34) *r* (*p* Value)
TAMs M2	0.20 (0.03)

**Table 4:** Relationship between TAMs M2, MVD (Mann–Whitney U test, * ANOVA Kruskal—Wallis test) and clinicopathological parameters.

Parameter		TAMs M2 Median (Mean)	*p* Value	MVD Median (Mean)	*p* Value
Age (median-62.5 years)	<62.5 ≥62.5	60 (77)	0.2	31 (34)	0.2
		56 (63)		28 (32)	
Gender	Female	60 (75)	0.9	34 (33)	0.4
	Male	57 (68)		29 (32)	
Depth of invasion (pT)	T1 (a + b)	30 (48)	* 0.04	25 (33)	* 0.2
	T2	68 (78)		25 (29)	
	T3	67 (74)		35 (34)	
	T4 (a + b)	48 (64)		29 (31)	
Regional lymph nodes (pN)	N (−)	46 (68)	0.3	30 (33)	0.8
	N (+)	60 (71)		30 (32)	
Stage (pTNM)	I (A + B)	35 (56)	* 0.06	25 (32)	* 0.5
	II (A + B)	58 (69)		28 (31)	
	III (A + B + C)	66 (77)		32 (34)	
	IV	32 (32)		30 (31)	
Histological type (Lauren)	Intestinal	54 (65)	* 0.3	31 (35)	* 0.5
	Diffuse	63 (76)		28 (31)	
	Mixed	68 (75)		29 (30)	
Location of tumor	Cardia	57 (70)	0.8	30 (32)	0.5
	Other location	60 (70)		30 (33)	

**Table 5:** The influence of selected clinicopathological parameters on overall survival (*n* = 106).

Parameter		Overall Survival Probability	*p* Value
TAMs M2	L (<58)	0.52	0.1
	H (≥58)	0.65	
MVD (CD34)	L (<30)	0.53	0.2
	H (≥30)	0.63	
Age (median-62.5 years)	<62.5	0.64	0.2
	≥62.5	0.53	
Gender	Female	0.69	0.1
	Male	0.54	
Depth of invasion (pT)	T1 (a + b) + T2	0.84	0.004
	T3 + T4 (a + b)	0.51	
Regional lymph nodes (pN)	N (−)	0.79	0.02
	N (+)	0.51	
Stage (pTNM)	I (A + B) + II (A + B)	0.70	0.04
	III (A + B + C) + IV	0.48	
Histological type (Lauren)	Intestinal	0.66	0.08
	Diffuse + Mixed	0.50	
Location of tumor	Cardia	0.59	0.7
	Other location	0.56	

## Data Availability

The raw data supporting the conclusions of this article will be made available by the authors on request.

## Abbreviations

The following abbreviations are used in this manuscript:

TAMs	Tumor-associated macrophages
CD 163	Cluster of differentiation 163
MVD	Microvessel density
GC	Gastric gancer
CD 34	Cluster of differentiation 34
pTNM	Pathological Tumor–Node–Metastasis
TME	Tumor microenvironment
TGF-β	Transforming Growth Factor β
AJCC	American Joint Committee on Cancer
HPFs	High-power fields
H&E	Hematoxylin and eosin
VEGF-C	Vascular Endothelial Growth Factor-C
VEGFR-3	Vascular Endothelial Growth Factor Receptor 3
ROC	Receiver operating characteristic
CD 105	Cluster of differentiation 105
EMT	Epithelial–mesenchymal transition
TECs	Tumor endothelial cells
TLRs	Toll-like receptors
IL-10	Interleukin-10
VEGF-A	Vascular Endothelial Growth Factor-A
CT	Computed tomography
CH25H	Cholesterol 25-hydroxylase
VM	Vasculogenic mimicry
